# Discovery of Novel Regulators of Autophagy in Animals

**DOI:** 10.1093/geroni/igaa057.2674

**Published:** 2020-12-16

**Authors:** Eric Baehrecke

**Affiliations:** UMass Medical School, Worcester, Massachusetts, United States

## Abstract

The clearance of organelles by autophagy is important for cell health, and defects in this process have been associated with age-associated degenerative disorders. Ubiquitination of proteins enables their recognition by cargo receptors that facilitate the delivery of both protein aggregates and organelles to forming autophagosomes for degradation. We have investigated developmentally programmed autophagy to identify novel regulators of organelle clearance. We identified an Atg7-independent autophagy program that is required for cell size reduction and clearance of mitochondria. We have used this system to screen for new factors regulate ubiquitin-dependent autophagy and clearance of organelles. We screened a collection of putative ubiquitin binding domain encoding genes, and identified the novel gene Vps13D. Vps13D is an essential gene that is necessary for autophagy, mitochondrial size, mitochondrial clearance, and is associated with human movement disorders. We have used genetics and biochemistry to identify factors that link Vps13D, mitochondrial biology and autophagy.

